# Fluctuation scaling in neural spike trains

**DOI:** 10.1186/1471-2202-15-S1-P119

**Published:** 2014-07-21

**Authors:** Shinsuke Koyama

**Affiliations:** 1 Department of Statistical Modeling, The Institute of Statistical Mathematics, Tokyo, Japan; 2 ERATO Sato Live Bio-Forecasting Project, Japan Science and Technology Agency, Kyoto, Japan; 3 Advanced Telecommunications Research Institute International (ATR), Kyoto, Japan

## 

The fluctuation scaling (FS) law has been observed in a wide variety of phenomena. It states that the variance of a quantity has a power function relation with the mean. Since Taylor found it in ecological systems [1], the FS law has been demonstrated in many natural and social systems, showing a universality of the law [2]. In this study, The FS law for neural spike trains is formulated using the framework of renewal point processes.

In order to quantify the variability of neural firing, two quantities: inter-spike interval (ISI) and spike count, are often measured from spike trains. We first consider the ISI statistics. Let $t_{1},t_{2},\ldots ,t_{n}$ be a sequence of spike times, and $x_{i}=t_{i}-t_{i-1}\left(i=2,\ldots ,n\right)$ be ISIs. Letμ=E(X) and $\sigma^{2}=Var\left(X\right)$ denote the mean and variance of ISI, respectively. We consider spike trains such that under a stationary condition $\sigma^{2}$ has a power function relation with  μ as

(1) $$\sigma^{2}=\phi \mu^{\alpha }.$$

The scaling exponent  α characterizes the 'intrinsic' dispersion of neuronal firing. For a Poisson (random) process, α=2. On the other hand, α>2(α<2) implies the tendency for the timing of spikes to be over (under) dispersed for large means, and under (over) dispersed for small means.

Consider next the counting statistics. Let $N_{\left(t,t+\text{Δ}\right]}$ be the number of spikes occurred in the counting window (t,t+Δ]. We prove that if the spike train is a renewal process and the interval statistics has the scaling property (1), then the variance of $N_{\left(t,t+\text{Δ}\right]}$ per unit time is asymptotically scaled by the mean of $N_{\left(t,t+\text{Δ}\right]}$ per unit time (i.e., the rate) as

(2) $$\frac{Var\left(N_{\left(t,t+\text{Δ}\right]}\right)}{\text{Δ}}=\phi {\left[\frac{E\left(N_{\left(t,t+\text{Δ}\right]}\right)}{\text{Δ}}\right]}^{\beta }$$

with β=3-α for Δ>>1.

In the presentation, I show the following two results:

The fluctuation scaling law emerges in the first-passage time to a threshold of certain diffusion processes (i.e., integrate-and-fire models).

The likelihood function of spike trains is constructed, based on which I propose a method for extracting the scaling exponent from nonstationary spike trains. This method is applied to biological spike train data to characterize the variability of neuronal firing.

Possible implications of these results are discussed in terms of characterizing intrinsic dynamics of neuronal discharge.
